# Small-scale regional engineering geological study of the Czech Republic evaluating the relationship between slope gradients and engineering geological zones

**DOI:** 10.1038/s41598-024-55972-z

**Published:** 2024-03-06

**Authors:** Marian Marschalko, Zofia Zięba, Kateřina Růžičková, Jan Růžička, Jan Kubáč, Jolanta Dąbrowska, David Sysala, David Krčmář

**Affiliations:** 1 Department of Geological Engineering, Faculty of Mining and Geology, VŠB-Technical University of Ostrava, 708 33 Ostrava, Czech Republic; 2 Department of Civil Engineering, Faculty of Environmental Engineering and Geodesy, Wrocław University of Environmental and Life Sciences, 50-365 Wrocław, Poland; 3 Department of Geoinformatics, Faculty of Mining and Geology, VŠB-Technical University of Ostrava, 708 33 Ostrava, Czech Republic; 4 Department of Hydrogeology, Faculty of Natural Sciences, Comenius University, Mlynská Dolina, 842 15 Bratislava, Slovak Republic

**Keywords:** Geology, Civil engineering

## Abstract

The aim of the small-scale regional engineering geological study of the Czech Republic was to evaluate the relationship between slope gradient and engineering geological zones. The research motivation was to determine the average slope gradient, 25%, 50% (median) and 75% quantiles related to the different engineering geological zones. This scientific information is critical from the perspectives of engineering geology, geotechnical engineering, and foundation engineering because an increasing slope gradient evokes the need to create a cut respectively foundation excavation or another excavation in the geological structure, which increases the probability of occurrence of the problem in terms of differential settlement and bearing capacity of the structures. The research was carried out in the territory of the Czech Republic in 8 Quaternary zones with soil foundation ground and 10 pre-Quaternary zones with rocks and semi-rocks and their eluvia. A significant difference in the statistical characteristics of slope gradients was found in the group of Quaternary engineering geological zones (evaluated group I) compared to the group of pre-Quaternary zones (evaluated group II). The value range of the average slope gradient was 1.65° (16.9%) to 5.89° (60.3%) for the Quaternary engineering geological zones (soil foundation ground), representing 43.4% difference. Whereas for the over-quaternary engineering geological zones (rocks, semi-rocks and their eluvia), the difference was much higher, 3.59° (36.8%) to 9.76° (100%—value determined as a referential because it was the maximum), which is also reflected in a more significant percentage difference of 63.2%.

## Introduction

An essential part of engineering geology is the knowledge of slope gradients (angles of slope^1–5^. There are many justifications for this statement. Thus, the study will further state examples. One of the most critical tasks of engineering geology is the description of geological engineering conditions for building structures within the civil engineering industry. The slope gradient is affected by a number of factors, such as erosion^6–8^, landslides^9–11^, neotectonic processes^12–14^ and others.

**Figure 1:**
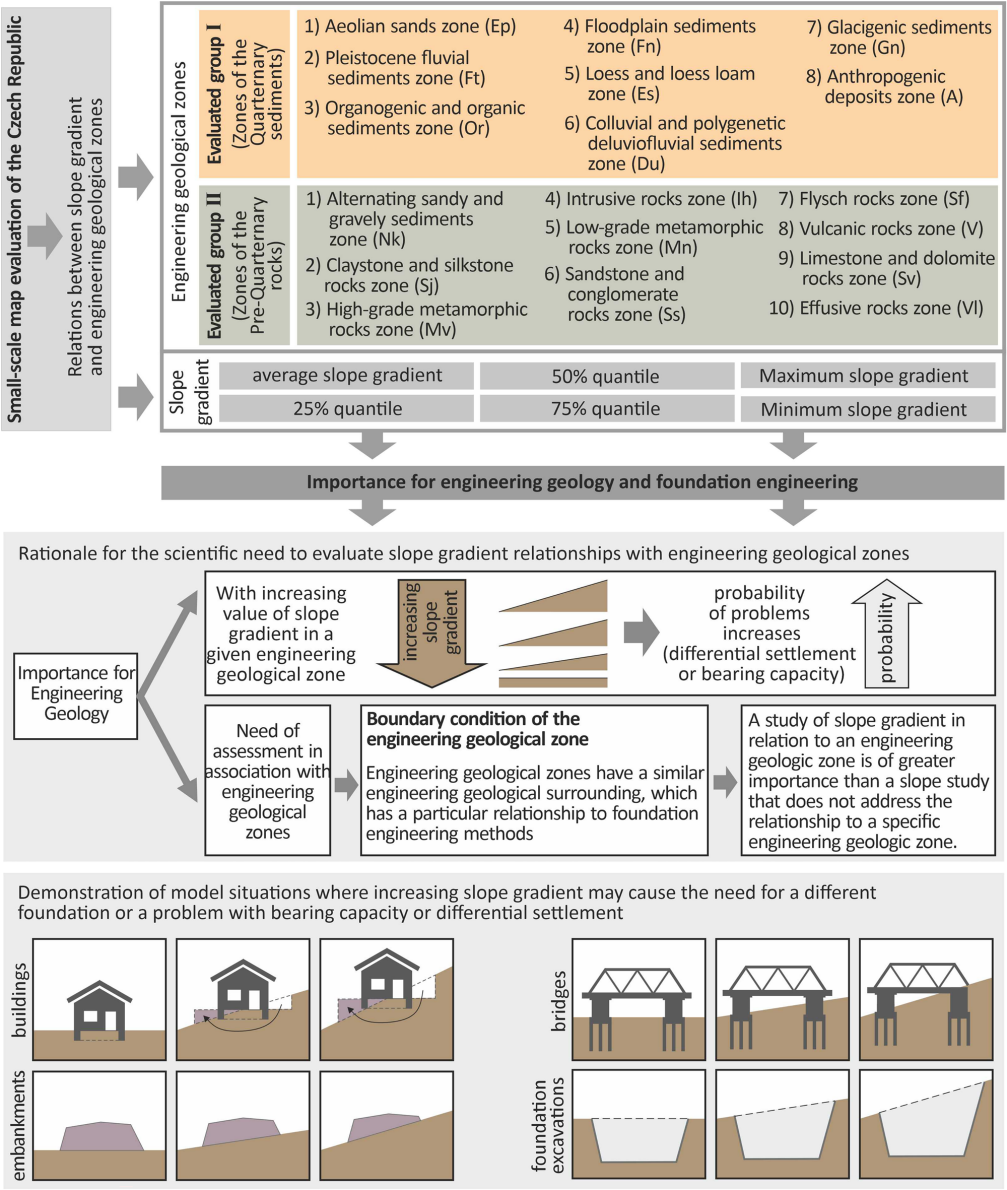
Schematic representation of the scientific objectives of the study.

When founding a structure, one of the most important boundary conditions is the slope gradient, which determines whether the structure will be implemented without a cut (open cut), (flat terrain), within a cut (sloping terrain), or combined partially within the cut^15–17^ and partly in an embankment (Fig. 1). Mentioning this example follows that it is very positive information (another scientific motivation of the study) to know the slope gradients (based on DMR—first boundary condition) in a specific engineering geological structure (second boundary condition), this is represented in the study by engineering geological zones^18–21^.

Understanding the engineering geological conditions is a key boundary condition for the study implementation, which is met by the engineering geological zoning maps^22–24^. These maps were created as a simplified model with similar engineering geological conditions for engineering geology, geotechnics and foundation engineering needs. Based on the combination of the two boundary conditions mentioned above, a study was developed to evaluate the relationship of slope gradient to engineering geological zones.

That stated, all statistically processed slope gradients were evaluated in each engineering geological zone, meaning in areas with similar engineering geological conditions^25–27^. This information will be needed for scientists in the fields of engineering geology, geotechnics and foundation engineering. Still, it will also be important didactically and for project designing activities (designer's idea of evaluating environments). Thus, new information about statistically processed data will be obtained within which they can calculate specific slope gradient values in the Czech Republic in terms of their ranges (minimums and maximums) in similar engineering geological environments (engineering geological zones).

In addition to the value of the variance, between the minimum and maximum, the statistical characteristics of the 25%, 50% (median), 75% quantile, and average slope gradient were investigated (Fig. 1). All these statistical values in each engineering geological zone allow to assume (calibrate assumptions in design activities) more frequent slope gradients in each engineering geological environment (zone). This means that we can work with more probable variations in terms of frequency.

The abovementioned statements give engineering geologists and geotechnical engineers a basic overview of slope gradients in each engineering geological zone. It is evident that the values obtained in this way can be worked with in a structured way because the more extensive engineering geological zones will be of much more practical importance than the less extensive ones. However, their variability, even in terms of less frequent types, will provide an interesting possibility to compare this important parameter (slope gradients).

Within the research, slope gradients will be studied within each engineering geological zone (Fig. 1). It should be understood that the engineering geological map is essentially a combination of a Quaternary map and an exposed geological map. An area where Quaternary geology exists is an area that is characterised by Quaternary engineering geological zones^28–30^. Within the Czech Republic, these are the following 8 engineering geological zones^20,30^. In particular, these zones include the Aeolian sands zone (Ep), Pleistocene fluvial sediments zone (Ft), Organogenic and organic sediments zone (Or), Floodplain sediments zone (Fn), Loess and loess loam zone (Es), Colluvial and polygenetic deluviofluvial sediments zone (Du), Glacigenic sediments zone (Gn) and Anthropogenic deposits zone (An).

In areas where the Quaternary geology is not developed or is developed in the form of eluvium, the area is characterised by pre-Quaternary engineering geological zones (Neogene to Neo-proterozoic)^30,31^. Within the Czech Republic, there are 10 of these^20,30^, and they include the Alternating sandy and gravely sediments zone (Nk), Claystone and siltstone rocks zone (Sj), High-grade metamorphic rocks zone (Mv), Intrusive rocks zone (Ih), Low-grade metamorphic rocks zone (Mn), Sandstone and conglomerate rocks zone (Ss), Flysch rocks zone (Sf), Vulcanic rocks zone (V), Limestone and dolomite rocks zone (Sv) and Effusive rocks zone (Vl).

The scientific need for slope gradient research in engineering geological zones has already been mentioned above. It is further necessary to state that as the slope gradient value in a given engineering geological zone increases, the probability of problems related to differential settlement^32,33^ or bearing capacity^34,35^ increases. Each engineering geological zone behaves individually according to the engineering geological conditions and according to the physical–mechanical parameters, especially in relation to compressibility (deformation modulus)^36–38^.

The study of slope gradient concerning the engineering geological zone is of greater significance than the study of a slope, not related to a specific engineering geological environment. A change in slope gradient combined with a particular adverse geological structure may necessitate a different foundation of the structure, bearing capacity or differential settlement problem. In principle, each foundation of different engineering structures has a varied relationship to changes in slope gradient. Figure 1 shows four schematic model situations related to buildings, embankments, bridges, and foundation excavations. The above fact should therefore be seen as an additional motivation for the study, as it is of great practical value. This value needs to be perceived in a structured way, both from a design perspective and a technical implementation and construction economics perspective.

## Materials and methods

### Implementation of the study in the area of interest in the Czech Republic

The digital terrain model (DMR4G—Digital Terrain Model), consisting of a matrix of points with 5 × 5 m spacing, was used as the basis for the slope gradient. Their height accuracy is given by a mean height error of 0.3 m in the exposed terrain. In the part of the DMR where the terrain was forested, the mean error was 1 m. As part of the pre-processing, this representation (matrix of points) was converted into a matrix of square cells that are 5 × 5 m in size. In the next stage, the slope was calculated by deriving the slopes from the relief rasters by calculating the slopes based on the analysis of the surroundings, i.e. using a convolutional floating window, where for each cell, the surroundings of a 3 × 3 cell were evaluated and from these 9 elevation data, the slope for the central cell was calculated. Subsequently, an overlay analysis of the slope layer and the engineering geology zone layer was performed. The engineering geological zones were represented by polygons with areas of similar engineering geological conditions. It is important to note that each engineering geological was not formed by only one polygon but by a set of polygons in different parts of the Czech Republic. Thus, all the statistical characteristics were calculated summarily for all polygons forming one type of engineering geological zone. The following statistical characteristics were calculated: average slope gradient, minimum and maximum slope gradient, 25% quantile, 50% quantile (median), and 75% quantile of slope gradient.

**Figure 2:**
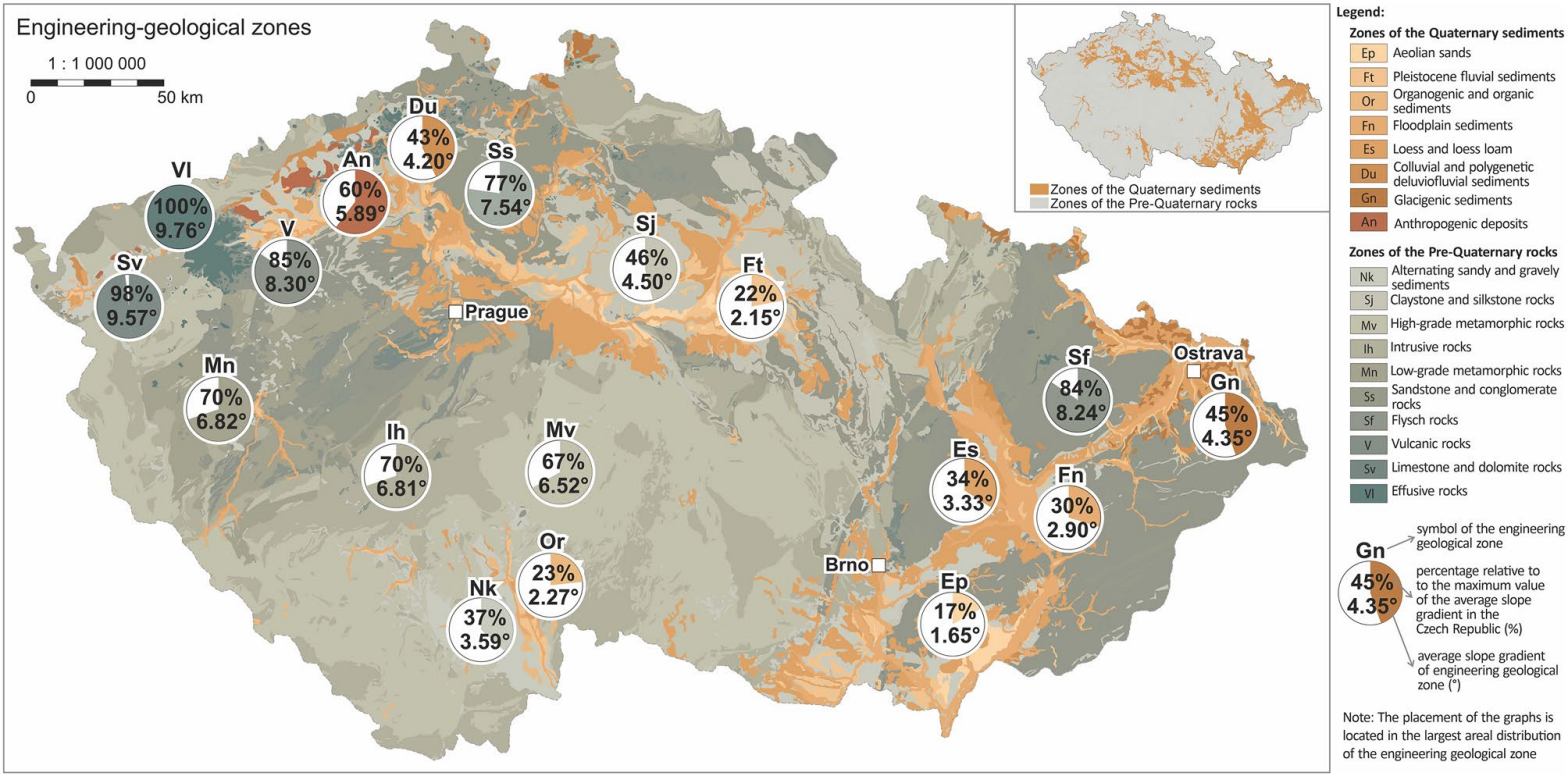
Regional map of engineering geological zones showing average slope gradients and their percentages in each engineering geological zones.

The study was carried out on the territory of the Czech Republic, where slope gradients in each engineering geological zone were evaluated. During assessing the study area in terms of the character of the zones, it is necessary to distinguish between two basic types (Fig. 2). The first type is the engineering geological zones with Quaternary geological structure with foundation ground of soil character. Their extent in the Czech Republic is 19.8%. These Quaternary engineering geological zones are located mainly in the eastern part of the Czech Republic in connection with the occurrence of the Carpathian foreland, which is covered with Quaternary sediments with these types of zones. The second major concentration of Quaternary zones is in the area of the Bohemian Cretaceous Table. The remaining Quaternary geological structure is already smaller in the area (Fig. 2).

The second type is the pre-Quaternary engineering geological zones (Fig. 2), representing the rest of the Czech Republic (excluding the first type of Quaternary engineering geological zones). Their share in the entire territory of the Czech Republic is 80.2%, which is a rather surprising figure for a single country.

On the map of engineering geological zones, a map marker (Fig. 2) is labeled with the name of the engineering geological zone and the value of the average slope gradient (also with the percentage value). Furthermore, a pie chart with its percentage in relation to the maximum average slope gradient within one engineering geological zone of the Czech Republic. When comparing Quaternary zones with the pre-Quaternary zones, it can be observed that their average slope gradients are lower.

### Visualisation of slope gradients in individual engineering geological zones of the Czech Republic

**Figure 3:**
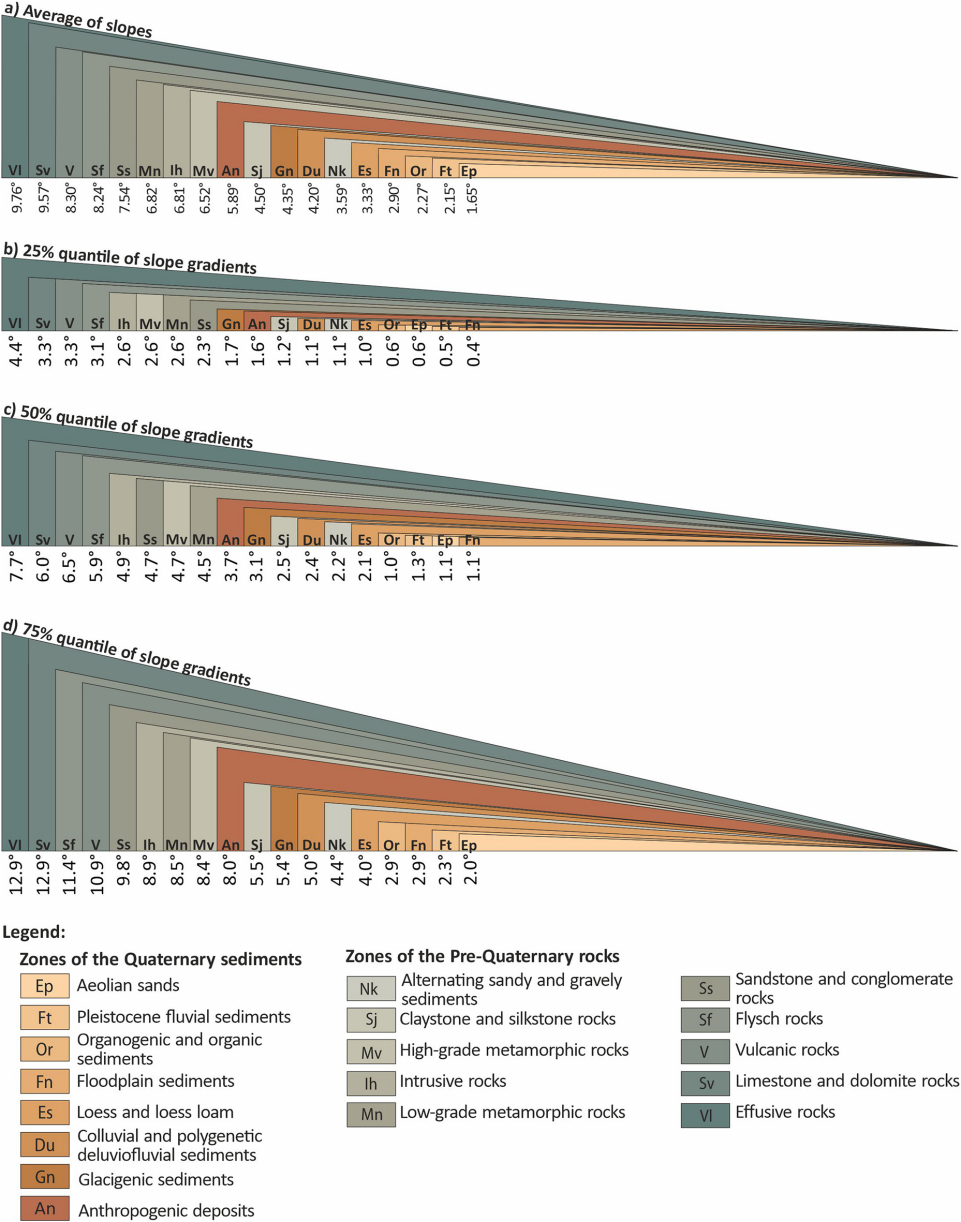
Visualisation of the actual slope gradients in each engineering geological zone of the Czech Republic: (**a**) Average of slopes, (**b**) 25% quantile of slope gradients, (**c**) 50% quantile of slope gradients, (**d**) 75% quantile of slope gradients.

Slope gradients in engineering geological zones obtain an important aspect which is their visualisation in the form of displaying unbiased actual slope gradients*.* This imaging approach allows to get an accurate and real idea of the shape of these slopes (Fig. 3). While evaluating this aspect, there is apparent that a distinction can be made between a group of zones that are made up of pre-Quaternary engineering geological zones, where the surface geology consists of rocks and semi-rocks and their eluvia (evaluated group II). These zones are shown in darker grey and grey-brown in the figure (Fig. 3). This group can be observed to have a higher slope gradient than the other group of zones made up of different shades of brown (Fig. 3). This group of zones is made up of quaternary engineering geological zones with foundation ground of soil character. The group of zones formed by rocks and semi-rocks and their eluvia has indisputably higher values of compressive strength but also shear strength, whereas the opposite is the case for the second group of soil zones (evaluated group I). The above properties are of major importance for slope gradients, although the slope gradient is multifactorial in nature and is not solely dependent on the above properties.

Although there is a fundamental difference in slope gradients between the two groups of engineering geological zones (evaluated group I and II), there are exceptions that do not meet a specific aspect of this group. Thus, they are located at the interface between the two groups and cause no sharp colour interface between the two groups in the assessed image, which is reflected by the fact that there is a particular mix of colours near the interface (Fig. 3).

The exceptions are the three engineering geological zones. The first of these (Fig. 3) is the Anthropogenic Deposits (An) zone, which belongs to the group of zones that are both Quaternary in age and composed of soil. However, what is specific about them, is that they are composed of grains of different sizes that have been extracted from greater depths. Also, they were rocks before extraction. Thus, after mining, their grains have a higher compressive strength than soils with other natural genetic types. At the same time, they also have pointed grains, which, combined with the previous property, causes them to have a higher shear strength and, consequently, a higher slope gradient.

The second zone (Fig. 3) specific in terms of slope gradient is the pre-Quaternary Alternating sandy and gravely sediments (Nk) zone. Most of the pre-Quaternary engineering geological zones are composed of diagenetically altered rocks, resulting in the fact that they are rocks or semi-rocks. In this instance, it is not the case because the listed sediments of this zone are relatively young (Neogene) and unconsolidated. Their position in terms of depth of deposition also contributes to this fact. The fact that they are unconsolidated causes their lower shear strength and, consequently, a lower slope value compared to other pre-Quaternary zones.

The third zone (Fig. 3), which has relatively different characteristics from the other pre-Quaternary zones, is the Claystone and Silkstone rocks (Sj) zone. The reason for the specificity of this zone is the fact that it has relatively lower values of compressive strength, which makes them more prone to erosion and, as a result, produces eluvia of greater strength than the other pre-Quaternary engineering geological zones of the Czech Republic. This combination of characteristics causes it to have lower slope gradient values than other zones in its group.

Comparing four selected statistical characteristics of slope gradients in terms of average slope gradient (Fig. 3a), 25% quantile (Fig. 3b), 50% quantile (Fig. 3c) and 75% quantile (Fig. 3d), it was found that the 75% quantile of the Effusive Rocks (Vl) pre-Quaternary engineering geological zone had the maximum value of 12.9° among all these views. That engineering geological zone reached the highest value for all the statistical characteristics evaluated (average slope, 25% quantile, 50% quantile, 75% quantile). We can state that the maximum value for the average slope gradient reached 9.76°, representing a difference of 3.14° compared to the maximum observed statistical value at the 75% quantile. Meanwhile, the maximum value for the 50% quantile was recorded at 7.7°, indicating a difference of 5.2°. The lowest value among the four statistical characteristics in Fig. 3 was noted for the 25% quantile, with 4.4°, signifying a difference of 8.5° compared to the compared maximum value (at the 75% quantile).

The fact that one zone formed the maximum value of slope gradient for all statistical characteristics does not apply to the minimum value. The minimum value was recorded for slope gradient in the two more critical statistical characteristics (average slope—Fig. 3a and 75% quantile—Fig. 3d) for the Quaternary soil engineering geological zone Aeolian sands (Ep). Whereas, for 25% (Fig. 3a) and 50% (Fig. 3b) quantile, the minimum value was recorded for the Quaternary soil engineering zone Floodplain sediments (Fn). The maximum value of the minimum slope gradient among the four statistical characteristics evaluated in Fig. 3 was recorded as 2.0° for 75% of the quantile (Fig. 3d). The minimum value of the average slope gradient (Fig. 3a; 1.65°) was lower by 0.35° compared to the minimum value at the 75% quantile. In contrast, the minimum value for the 50% quantile (Fig. 3c; 1.1°) was lower by 0.9°, and the minimum value for the 25% quantile (Fig. 3a; 0.4°) was lower by up to 1.6°.

### Ethical approval

This article does not contain any studies with human participants or animals performed by any of the authors.

## Results and discussion

### Evaluation of slope gradients in engineering geological zones of the Czech Republic in relation to landscape elements

**Figure 4:**
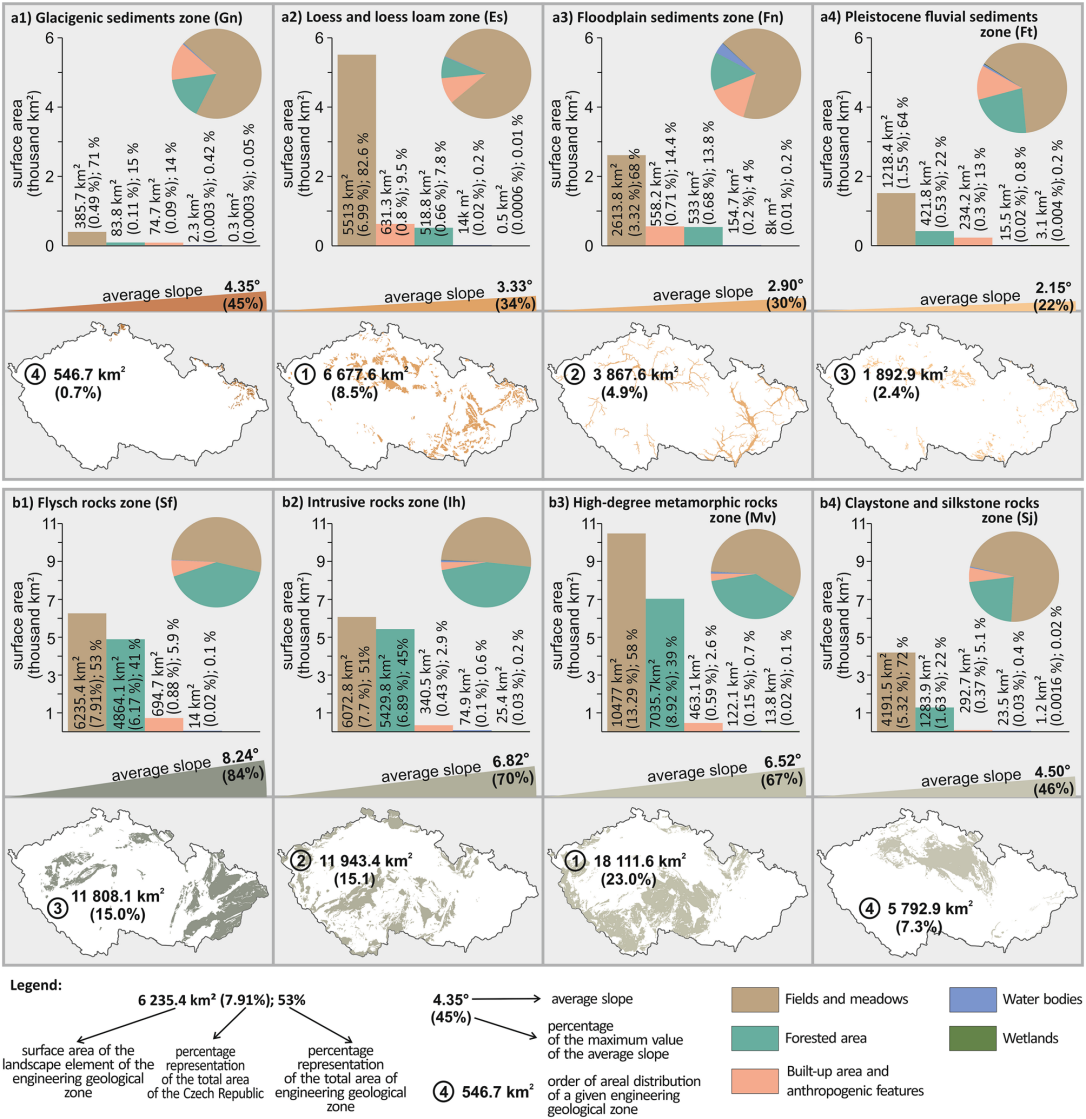
Bar and pie charts of the area distribution of individual landscape elements within each engineering geological zone of the Czech Republic in two groups, where the evaluated group I is of Quaternary engineering geological zones with soil foundation ground (**a1**–**a4**), the evaluated group II is of the 5 largest pre-Quaternary engineering geological zones with rocks and semi-rocks and their eluvia (**b1**–**b4**): (**a1**) Glacigenic sediments zone (Gn), (**a2**) Loess and loess loam zone (Es), (**a3**) Floodplain sediments zone (Fn), (a4) Pleistocene fluvial sediments zone (Ft), (**b1**) Flysch rocks zone (Sf), (**b2**) Intrusive rocks zone (Ih), (**b3**) High-degree metamorphic rocks zone (Mv), (b4) Claystone and silkstone rocks zone (Sj).

In addition to the slope gradient in the engineering geological zones, the study also evaluated the influence of landscape elements. Evaluation groups I and II were selected for this assessment. The evaluated group I had the four most extensive engineering geological zones with soil foundation ground (Fig. 4). The evaluated group II consisted of the four most extensive engineering geological zones with rocks and semi-rocks and their eluvia. Comparing these two evaluated groups shows that group II (rocks and semi-rocks and their eluvia) evidently has a more extensive area distribution in the Czech Republic than group I (soil masses). The High-degree metamorphic rocks zone (Mv) has the most extensive area distribution with 18 111 km^2^, which has a 23% share in the whole territory of the Czech Republic. At the same time, the most extensive area of the Loess and loess loam zone (Es) has only 6 677 km^2^ (8,5% of the Czech territory).

It was found that there is a considerable variation between the slope gradients in the two groups of zones evaluated, which also affects the nature of the landscape elements in these zones. In the evaluated group II of rocks and semi-rocks, the Flysch rocks zone (Sf) has the highest average slope with 8.24° (Fig. 4). Whereas, in the evaluated group I of soil masses, it is the Glacigenic sediments zone (Gn) with about half of 4.35°. As far as the minimum slope is concerned, it is similar. That is, for the rock and semi-rock group, the average slope is 4.50° for the Claystone and silkstone rocks (Sj), and for the soil masses, it is only 2.15° for the Pleistocene fluvial sediments zone (Ft).

This large change in slope gradient in both groups evaluated is related to the change in the nature of the landscape elements. The most significant change found between the evaluated groups is that the evaluated group II (rock and semi-rock areas with their eluvia) has a significantly higher proportion of the landscape element Forrested area (with an area range of 22–45%) than the evaluated group I of soil areas (only a range of 7.8–22%). One of the reasons for this is the importance of slope gradient, as the evaluated group I has higher slope gradients allowing forestry activities to be carried out more economically and technically than agricultural activities. Therefore, it is often preferred. The forestry activity does not require frequent travelling over the terrain, as is the case with agricultural activity, where it is necessary to drive the equipment several times a year. Thus, the terrains (landscapes) with higher gradients may be more suitable for forestry activities than agricultural ones. A further reason is the importance of the nature of the foundation ground in both of these evaluated groups. The evaluated group II has less suitable physical–mechanical properties in the cultivation of agricultural soils (a higher proportion of rock fragments in the soils due to the existence of a weathering mantle in the environment of rocks and semi-rocks) than the evaluated group I of soil zones. This fact also leads in many areas to lower economic profitability of agricultural activity compared to forestry.

In both groups, the most extensive landscape element is fields and meadows. In the evaluated group I of soil zones, this landscape element is completely dominant and has higher values of area coverage than 64%. In contrast, in the evaluated group II, the area coverage of this landscape element is below this value. There is only one exception, namely the Claystone and Silkstone rocks zone (Sj), as this area has lower values in compressive strength and, at the same time, a greater thickness of eluvium than the other areas of this group. In general, when evaluating the occurrence of the landscape element fields and meadows in relation to slope gradients, it can be concluded that the areas with lower slope values can be more easily cultivated with machines. At the same time, these engineering geological zones have the character of soil foundation ground, which have more suitable properties than rocks and semi-rocks and their eluvia. The result points out a greater economic advantage for farming and, therefore, a higher percentage of this landscape element.

Suppose assessing the proportion of built-up area and anthropogenic features. In that case, there is a difference between the evaluated groups I and II of the zone in that the proportion is much higher in the evaluated group I of land masses than in the evaluated group II. This information is reflected in the share of the first group in the total area of the zone ranging from 9.5 to 14%, whereas in the second group, the share of this landscape element ranges from only 2.6–5.9%. The reasoning underlies the fact that building is much more technically and economically feasible in low-lying areas with a lower slope than the other way around, which is characteristic of the evaluated group I of soil foundation ground zones. In flatter areas, there are lower costs for building objects because they can be constructively simpler. At the same time, we have lower earthwork costs in terms of excavation and embankment volume, and more suitable soil and rock workability (breakage resistance or mineability of rock/soil). Reflecting points out in the lower grades of workability, which are typical of soil masses. Whereas for the group of zones of the Pre-Quarternary rocks, we have less suitable workability. Thus, there are higher grades of workability, which ultimately means that the technical implementation of the countries of work is more complex, resulting in higher costs for the implementation of the structure.

### Statistical evaluation of the slope gradient in individual engineering geological zones of the Czech Republic

**Figure 5:**
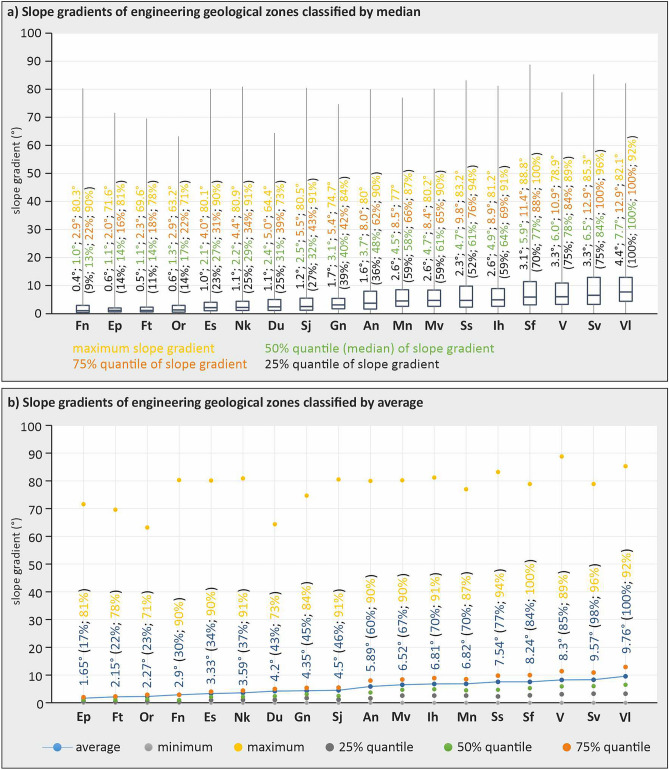
Graphs of slope gradient values in individual engineering geological zones of the Czech Republic: (**a**) Boxplot—scatter plot of values between the minimum quantile value (25% quantile) and the maximum quantile value (75% quantile) at the same time in the boxplot is shown a line that expresses the value of the median (50% quantile), (**b**) Display of values of minimum and maximum slope gradients, average slope gradient, 25, 50, 75% quantile.

The statistical evaluation of the slope gradient in the different engineering geological zones of the Czech Republic was evaluated using two basic graphs showing the results of the study (Fig. 5). The first evaluation of the study was performed using a boxplot, which includes a box plot of the scatter of values between the minimum quantile value (25% quantile) and the maximum quantile value (75% quantile), (Fig. 5a). At the same time, the boxplot shows a line that represents the median value (50% quantile). The great advantage of using the quantile is that it shows the probability distribution of the slope.

Supposing comparison of the difference in values between the 25% and 75% quantile is reflected graphically in the dimension, the height of the boxplot (Fig. 5a). To further compare the above difference (variance, boxplot) with each other, it is notable that it divides into three groups. The first group of the lowest differences (boxplots with the smallest dimension, with the smallest height) belongs to the group of 5 engineering geological zones with Quaternary geological structure and soil composition of the foundation ground. The second group of the most significant differences (boxplots with the highest dimension, with the greatest height) includes 8 engineering geological zones with pre-Quaternary geological structuring consisting of rocks and semi-rocks and their eluvia.

The transition group between the first and second groups comprises 5 engineering geological zones from each of these groups, with each zone having a particular characteristic that either decreases or increases the value of this variance. However, only 3 of these zones deviate from the observed trend of both groups. Of the Quaternary group (soil zones), this is the Anthropogenic Deposits zone, which consists of grains originating from mineral extraction (mainly coal). As a result, this group has a higher shear strength and also a higher slope gradient. Of the pre-Quaternary group, the Claystone and siltstone rocks are the most important. This zone is composed of softer rocks with lower compressive strength and is more prone to weathering and greater eluvial thickness. As a result, they have a lower slope gradient compared to other pre-Quaternary zones. Another engineering geological zone of this type is the Alternating sandy and gravely sediments zone, which has a lower shear strength because it is diagenetically unconsolidated due to its young age (Neogene) and has the character of soil foundation ground. The resulting then follows its lower slope gradient than other pre-Quaternary areas with rocks and semi-rocks (Fig. 5a).

The second evaluation of the study was carried out using a graph showing the minimum, maximum and average values as well as the 25%, 50% and 75% quantile of slope gradient of each engineering geological zone in the country (Fig. 5b). When evaluating the observed trends from the above graph, the following relationship is notable. As the slope gradient value increases in all of the above statistical characteristics except for the maximum slope gradient, the first trend can be observed that with increasing slope gradient value, the character of engineering geological zones gradually changes from soils (Quaternary geological structure) to rocks and semi-rocks (pre-Quaternary geological structure).

The second trend observed is that the slope gradient increases with increasing shear strength in the soil engineering geological zones. Although shear strength measurements within engineering geologic zones are not part of this study, this is a well-known fact. For example, zones with eolian sediments (Ep) have lower shear strengths than zones with glacigenic sediments or deluviofluvial sediments. Therefore, the shear strength increases with increasing grain size in the genetic type of zones.

The third trend is an increase in slope gradient with rising compressive strength in the evaluated group II of engineering geological zones with rocks and semi-rocks. At the same time, the thickness of eluvium decreases with increasing compressive strength. However, this is also influenced by age and erosion. Although compressive strength measurements were not part of the study, it is a well-known fact that engineering geological zones with genetic types of sedimentary rocks have lower compressive strengths than genetic types of zones with metamorphosed and igneous rocks. This well-known fact was confirmed in the present study by the related increase in slope (Fig. 5b), with the availability of specific quantification values in each regional unit of the engineering geological zones.

**Figure 6:**
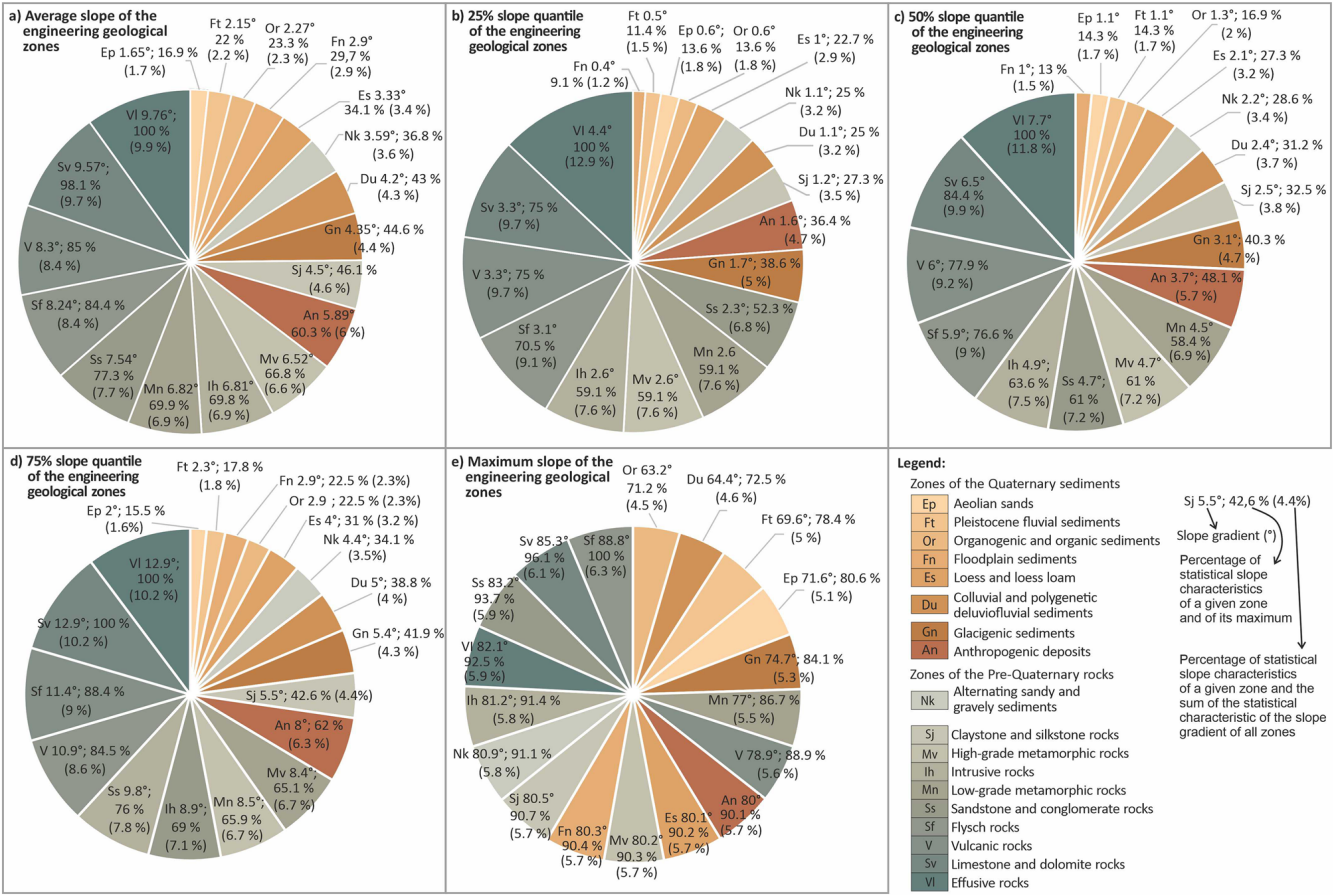
Pie charts of the percentage representation of statistical characteristics of slope gradients of individual engineering geological zones within the Czech Republic: (**a**) Average slope, (**b**) 25% slope quantile, (**c**) 50% slope quantile, (**d**) 75% slope quantile, (**e**) Maximum slope.

The percentages of each statistical characteristic (Fig. 6) of slope gradients were compared within each engineering geological zone. Significant percentage differences between the soil (Quaternary) zones and the rock and semi-rock (pre-Quaternary) zones are evident for all statistical characteristics (mean—Fig. 5a, 25% quantile—Fig. 6b, 50% quantile—Fig. 6c and 75% quantile—Fig. 6d) except maximum slope gradients (Fig. 6e). The maximum slope gradient values are broadly similar. Hence, the percentage representation of each zone is roughly similar. Thus, this statistical characteristic is unsuitable for assessing differences in slope gradients between the different engineering geological zones. For all other statistical characteristics, differences between Quaternary and pre-Quaternary engineering geological zones are visible.

When comparing the percentages of each statistical characteristic, it is certain that the greatest similarity in regards to the structure of the pie charts is between the 25% and 50% quantile and also between the 75% and the average value (Fig. 6).

**Figure 7:**
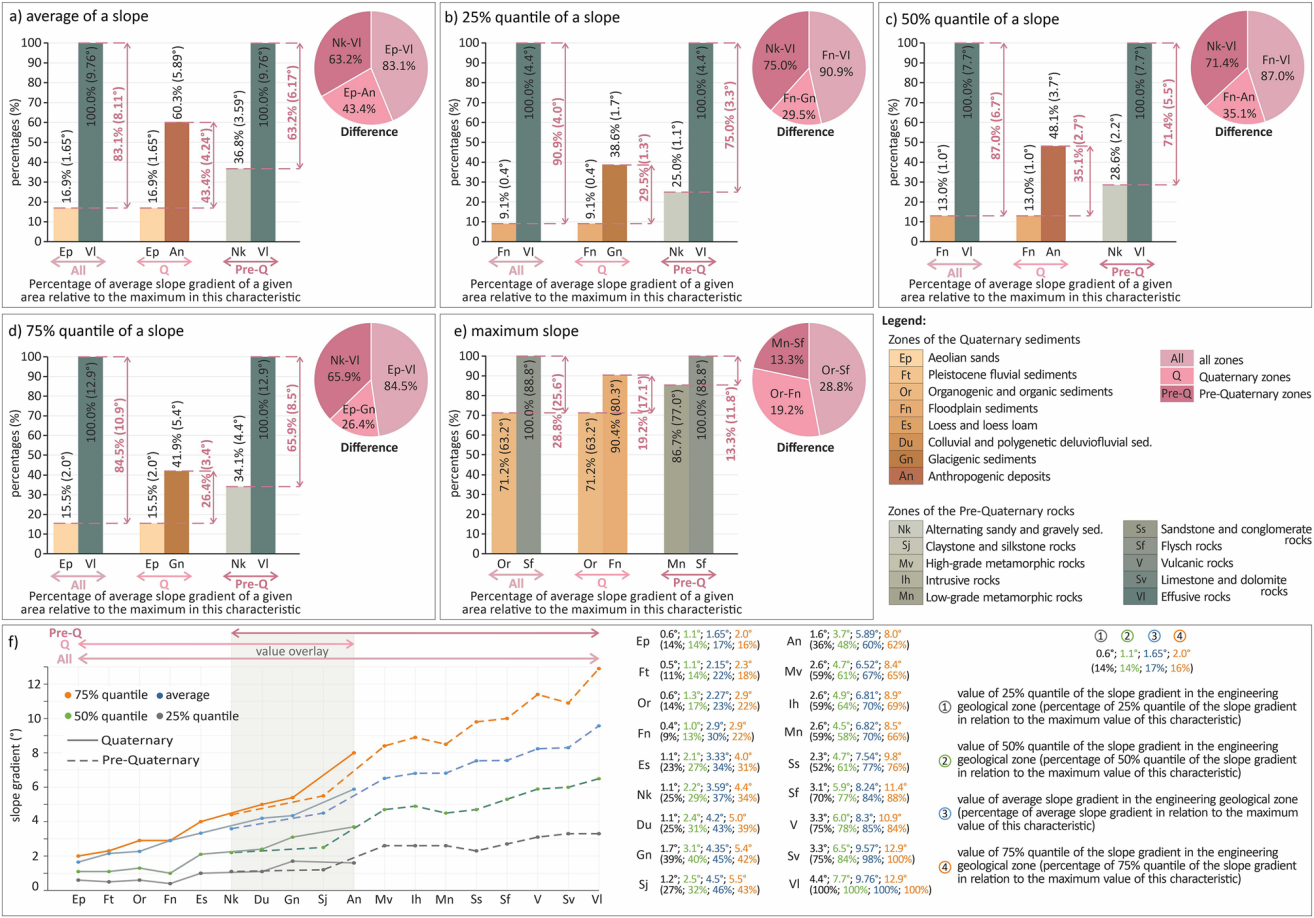
Graph of values of differences between minimum and maximum slope gradients in different statistical characteristics: (**a**) Average of a slope, (**b**) 25% quantile of a slope, (**c**) 50% quantile of a slope, (**d**) 75% quantile of a slope, (**e**) Maximum slope, (**f**) Plot of values of the statistical characteristics shown in (**a**–**d**), the values of the statistical characteristic in (**e**) are shown in Fig. 5b.

An essential result of the study from the scientific point of view is the finding of the difference between the minimum and maximum values of each statistical characteristic in the two groups (evaluated groups I and II) of engineering geological zones evaluated and also overall (Fig. 7).

As for the average slope gradients, it was found that the overall difference between the minimum and maximum values was 8.11°, which represents an 83.1% proportion relative to the maximum value of one engineering geological zone (Fig. 7). As far as the evaluated groups I and II are concerned, it is evidently notable that the slopes of the slopes in the Quaternary engineering geological zones with soil foundation ground have a lower average slope difference between the minimum and maximum values (4.24°–43.4%). This variation results from the Aeolian sands zone (1.65°–16.9%) and the Anthropogenic deposits zone (5.89°–60.3%), whereas the pre-Quaternary zones have a more significant difference (6.17°–63.2%), as they are mainly located in rocks and semi-rocks. This difference concerns the Alternating sandy and gravely sediments zone (3.59°–36.8%) and the Effusive rocks zone (9.76°–100.0%). These values than indicate that the difference between these two groups is up to 19.8% (1.93°).

When comparing the results of the difference in values (Fig. 7) between the statistical characteristics, we can basically see that the trends are similar between the four statistical characteristics (average slopes—Fig. 7a, 25%—Fig. 7b, 50%—Fig. 7c, 75% quantile of slope slopes—Fig. 7d). In contrast, the statistical characteristic of maximum slope gradient has a different trend because the difference between the values of this characteristic is significantly smaller (Fig. 7e). There is a greater similarity existing in the group of pre-Quaternary engineering geological zones (evaluated group II) with rocks and their eluvia. The above is manifested by the fact that all minima and maxima of the four statistical characteristics are formed by the same engineering geological zone. The minima are formed by the Alternating sandy and gravely sediments zone (Nk), and the maxima are formed by the Effusive rocks zone (Vl). This trend can be evaluated as more significant because this group's physical–mechanical properties behave more constantly (there are more differences between the properties of the engineering geological zones) in terms of slope changes.

Contrastingly, within the group of Quaternary engineering geological zones (evaluated group I) with soil foundation ground, there is a greater diversity of genetic types, where twice the Aeolian sands zone (Ep), Floodplain sediments zone (Fn) were the minima and twice the Anthropogenic deposits zone (An), Glacigenic sediments zone (Gn) were the maxima. Therefore, this evaluated group has less behavioural similarity compared to the evaluated group II than evaluated group I. In comparison, the remaining zones were already formed by other engineering geological zones. Based on this information, it can be argued that the physical–mechanical properties of the engineering geological zones of the soil types have more similar ranges of values (there are fewer differences between the properties of the engineering-geological zones compared to the evaluated group I), which causes more similar behaviour in slope formation. Consequently, a manifestation of greater diversity follows in their representation. Evidently, the slope formation is multifactorial in nature, but this does not preclude the previous description of behaviour.

## Conclusion

Within the study, a relationship was found in the average slope gradient in the engineering geological zones which gradually increases towards the individual soil engineering geological zones (Quaternary—evaluated group I) to the rocks and semi-rocks (pre-Quaternary—evaluated group II). This trend was also found in the other statistical characteristics assessed (25%, 50% and 75% quantiles). Thus, it means that the rock and semi-rock engineering geological zones (evaluated group II) always have a higher slope in the statistical characteristics than the soil engineering geological zones (evaluated group I).

When comparing the two evaluated groups in terms of similarity of behaviour (trend) in the different statistical characteristics evaluated, it can be seen that the evaluated group II had a higher-ranking similarity in relation to the increase in slope changes. At the same time, the evaluated group II has a higher range of values between the minimum and maximum values than the evaluated group I in each of the evaluated statistical characteristics (average slope, 25%, 50%, 75% quantile of slope gradients). This fact is related to the nature of the foundation ground in the pre-Quaternary engineering geological zones, as they are composed of rocks and semi-rocks and their eluvia. Thus, it means that the range of values of physical–mechanical properties of these types of foundation ground is much larger, which predisposes a higher difference in slope gradients.

Moreover, while comparing the evaluated groups I and II in terms of the occurrence of landscape elements, it was found that the areas with higher slope gradients (evaluated group II) have a higher proportion of the landscape element forests than the areas with lower slope gradients (evaluated group I). In addition, the evaluated group I of soil engineering geological zones with lower slope gradients has a higher proportion of the landscape elements fields, meadows and built-up areas than the evaluated group II with higher slope gradients. The relationship is related to the fact that a specific type of foundation ground in combination with the slope gradient predisposes a certain advantage or disadvantage (technical, economic, environmental) for a particular engineering geological zone in terms of the presence of a landscape element.

The information obtained from the study on slope gradients of engineering geological zones will help scientists, engineering geologists, geotechnical engineers, designers and builders to get an idea of slope gradients in the Czech Republic in relation to individual zones. In particular, this will help them to anticipate certain problematic issues with differential settlement concerning the nature of the engineering geology in the individual zones assessed. A higher slope gradient means a greater probability of an issue with differential settlement, but only for a particular geological structure (the engineering geological zones provide fundamental information on the engineering geological conditions). The study will provide some introductory information which will then be resolved in the context of a local engineering geological investigation at the location. Addressing this issue is one of the most important topics for engineering geologists and geotechnical engineers.

## Data Availability

All data used and analysed during the current study available from the corresponding author on reasonable request.
